# A randomised crossover study to compare the cross-sectional and longitudinal approaches to ultrasound-guided peripheral venepuncture in a model

**DOI:** 10.1186/s13089-017-0064-1

**Published:** 2017-04-03

**Authors:** James Griffiths, Amadeus Carnegie, Richard Kendall, Rajeev Madan

**Affiliations:** 1 School of Clinical Medicine, University of Cambridge, Cambridge, UK; 2 Emergency Department, Cambridge University Hospitals NHS Foundation Trust, Cambridge, UK; 3 Robinson College, Grange Road, Cambridge, CB3 9AN UK

**Keywords:** Ultrasound, Ultrasound education, Venepuncture, Emergency medicine, Emergency ultrasound, Ultrasound guided

## Abstract

**Background:**

Ultrasound-guided peripheral intravenous access may present an alternative to central or intraosseous access in patients with difficult peripheral veins. Using venepuncture of a phantom model as a proxy, we investigated whether novice ultrasound users should adopt a cross-sectional or longitudinal approach when learning to access peripheral veins under ultrasound guidance. This result would inform the development of a structured training method for this procedure.

**Methods:**

We conducted a randomised controlled trial of 30 medical students. Subjects received 35 min of training, then attempted to aspirate 1 ml of synthetic blood from a deep vein in a training model under ultrasound guidance. Subjects attempted both the cross-sectional and longitudinal approaches. Group 1 used cross-sectional first, followed by longitudinal. Group 2 used longitudinal first, then cross-sectional. We measured the time from first puncture of the model’s skin to aspiration of fluid, and the number of attempts required. Subjects also reported difficulty ratings for each approach. Paired sample *t*-tests were used for statistical analysis.

**Results:**

The mean number of attempts was 1.13 using the cross-sectional approach, compared with 1.30 using the longitudinal approach (*p* = 0.17). Mean time to aspiration of fluid was 45.1 s using the cross-sectional approach and 52.8 s using the longitudinal approach (*p* = 0.43). The mean difficulty score out of 10 was 3.97 for the cross-sectional approach and 3.93 for the longitudinal approach (*p* = 0.95).

**Conclusions:**

We found no significant difference in effectiveness between the cross-sectional and longitudinal approaches to ultrasound-guided venepuncture when performed on a model. We believe that both approaches should be included when teaching ultrasound-guided peripheral vascular access. To confirm which approach would be best in clinical practice, we advocate future testing of both approaches on patients.

**Electronic supplementary material:**

The online version of this article (doi:10.1186/s13089-017-0064-1) contains supplementary material, which is available to authorized users.

## Background

Achieving intravenous (IV) access is one of the most common, and most important, clinical skills used in hospital medicine. In the majority of patients, such vascular access is provided by a peripheral intravenous (PIV) cannula. Traditionally, PIV access is achieved by a landmark-and-palpation approach; however, locating a suitable vein by this method is not always straightforward.

Finding and accessing a peripheral vessel in patients with a history of IV drug abuse, severe obesity or chronic medical problems can be impossible using the landmark-and-palpation approach alone. Failure rates for this skill range from 9 to 56% [1]. The alternative in such cases is commonly a central line insertion.

While effective, central venous access is not without risks and is often inappropriate for patients requiring only a short duration of IV treatment. Catheter-related sepsis, arterial puncture, pneumothorax and arrhythmias are all recognised complications of central venous access, and can cause significant morbidity and mortality for these patients [2].

The increasing portability of ultrasound machines has led to a surge in the availability of point-of-care ultrasound (PoCUS) in the hospital environment. Ultrasound-guided central venous access is now considered the standard of care, improving both success rates and reducing complications such as arterial puncture [3]. It has been suggested that the use of ultrasound to guide the cannulation of the deep brachial or basilic veins in patients with difficult peripheral access may improve success rates and, ultimately, patient outcomes [4, 5].

Ultrasound-guided peripheral vascular access is a safe alternative to central line placement and could be used instead in up to 85% of patients classified as having difficult IV access [6]. Training students and staff to locate and access deep peripheral veins under ultrasound guidance may therefore lead to fewer central line placements and, consequently, a reduced incidence of central line-related complications.

There are two commonly used approaches for ultrasound-guided PIV access: the cross-sectional approach (short-axis or out-of-plane approach) and the longitudinal approach (long-axis or in-plane approach). Previous studies have shown that it is possible to teach this skill to novice ultrasound users effectively, using a single 30-min didactic training session [7, 8]. It is unclear as to whether novices find it easier to learn and effectively perform the cross-sectional approach or the longitudinal approach, and there is no consensus which approach should be taught. We devised this trial to identify which approach (or approaches) ought to be taught to novices and to help inform the development of a structured learning package.

We chose to conduct a prospective randomised controlled trial using medical students in order to answer this question. It has been demonstrated that previously ultrasound-naïve medical students are able to use ultrasound to guide peripheral vascular access on real patients after a single training session [9]. It has also been demonstrated that simulation-based training using a dedicated ultrasound phantom can lead to improved levels of skill when applied to real clinical practice [10]. However, we appreciate that we did not follow up phantom training with testing on patients. As such, we advocate testing with patients in future.

As we were testing the efficacy of novices, we used phantom models to simulate patients. Due to the construction of these phantom models, cannula lines tended to kink when inserted; thus, failure reflected the model limitations rather than the skill of the novice. To overcome this, we instead aimed for venepuncture, with the students required to aspirate 1 ml of fluid. This gave the benefit of requiring the students to correctly identify, locate and puncture the vein, while removing any failure bias due to the firm material of the model. We feel that as the main purpose of ultrasound guidance is to aid in locating and puncturing the vein, venepuncture acted as an adequate proxy for peripheral venous access.

## Methods

### Study design

We conducted a prospective randomised controlled trial to compare the effectiveness of the cross-sectional and longitudinal approaches to ultrasound-guided peripheral venepuncture when employed by novice ultrasound users. The study was reviewed and approved by the Cambridge Psychology Research Ethics Committee. All subjects gave written, informed consent before taking part in the study.

### Study setting and population

We recruited a convenience sample of 30 senior medical students at Cambridge University. Six sessions were held over the course of 2 weeks. There were five places available per session, and students were free to choose which session they attended. None of the students had used ultrasound to guide vascular access or venepuncture prior to this study. Nine of the 30 students reported having previously handled an ultrasound machine. All 30 of the students reported having at least some ultrasound teaching in the past.

### Study protocol

Each subject was randomly assigned to either the ‘cross-sectional first’ group (*n* = 14) or the ‘longitudinal first’ group (*n* = 16) prior to attending a session. This randomisation was performed according to the table generated by ‘Research Randomizer’, an online random assignment programme [11].

At each session, the group of five students was given a 30-min structured didactic teaching session in the form of an oral presentation. All teaching sessions were delivered by the same individual and covered:

- The value of ultrasound guidance in patients with difficult peripheral access;
- The basic physics behind ultrasound;
- Operation of the portable ultrasound machine;
- Interpretation of the image;
- A tutorial on how to use ultrasound to guide peripheral venepuncture.

Both the cross-sectional and longitudinal approaches were covered during this teaching session, and each approach was given equal coverage. A video walkthrough at the end of the presentation covered both approaches to the technique.

After the teaching presentation, all subjects were given an opportunity to practise using a Toshiba Viamo portable ultrasound machine to scan the blood vessels in the upper limbs of a human model. Subjects were taught how to operate the machine, and how to discriminate arteries from veins. Each subject was allowed 5 min practice with the machine and was asked to practise visualising vessels in both cross-sectional and longitudinal planes. Subjects were allowed to observe one another during this practice opportunity.

Subjects then underwent the study alone with the study coordinator. They were asked to use a 21-gauge needle and syringe to aspirate 1 ml of synthetic blood from a Blue Phantom (Kirkland, WA, USA) ultrasound training model under ultrasound guidance (Additional file 1). Subjects randomised to the ‘cross-sectional first’ group were required to use only the cross-sectional approach at this stage (Additional file 2). Likewise, the subjects randomised to the ‘longitudinal first’ group were required to use only the longitudinal approach for this task (Additional file 3). After succeeding, each subject was given a new needle and required to aspirate another 1 ml of synthetic blood from the model, this time using the approach that they had not tried previously. This crossover study design was employed to control for the effect of practice on performance.

### Measures

**Fig. 1:**
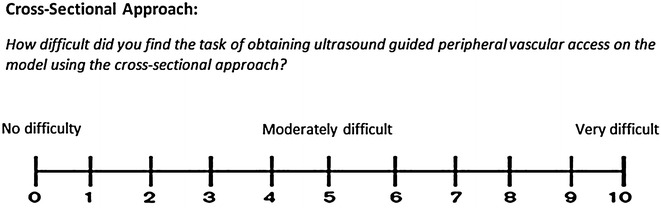
11-point visual analogue scale to assess and quantify how difficult subjects found the task of aspirating 1 ml of synthetic blood using the cross-sectional approach

**Fig. 2:**
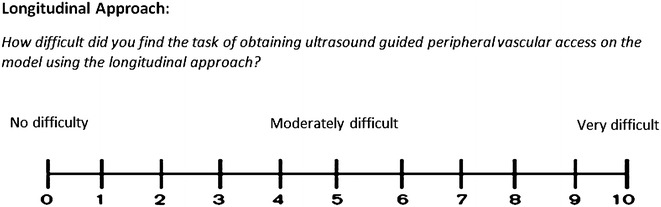
11-point visual analogue scale to assess and quantify how difficult subjects found the task of aspirating 1 ml of synthetic blood using the longitudinal approach

The time that elapsed from first puncture of the model’s skin to successful aspiration of 1 ml of synthetic blood into the syringe was measured. Also recorded was the number of attempts required successful aspiration i.e. the total number of times the needle passed through the model’s skin before aspiration of fluid. After attempting both approaches, subjects were asked to rate the difficulty of each approach using a pair of 11-point visual analogue scales (Figs. 1 and 2).

### Data analysis

Paired sample *t*-tests with 95% confidence intervals (95% CI) were used for the statistical analysis. Data were analysed using IBM SPSS Statistics 21 (IBM Corp., Armonk, NY, USA) [12]. Three separate paired sample *t*-tests were used to compare the cross-sectional and longitudinal approaches in terms of

- Mean time required;
- Mean number of attempts required;
- Mean difficulty rating.

Students were said to have ‘failed’ if more than three minutes had elapsed between first pass of the needle and aspiration of the fluid, or if three or more attempts were required. Students exceeding three needle passes were assigned the maximum value of three attempts, and students exceeding 3 min were assigned the maximum value of 180 s. This was decided on in advance, and was done for the purpose of statistical analysis in order to prevent excessive skewing of results by outliers.

## Results

The data for all 30 subjects were analysed. No student was excluded from the data analysis.

The mean time from first puncture of the model’s skin to successful aspiration using the cross-sectional approach was 45.1 s (95% CI 29.4 to 0.9 s). The mean time required using the longitudinal approach was 52.8 s (95% CI 36.7 to 69.0). Performing a paired samples *t* test, we found that the difference of 7.7 s (95% CI −12.1 to 27.4) was not statistically significant (*p* = 0.43).

The mean number of skin punctures required using the cross-sectional approach was 1.13 (95% CI 0.94 to 1.32). The mean number of skin punctures required with the longitudinal approach was 1.30 (95% CI 1.08 to 1.52). Paired sample *t*-tests demonstrated that this difference of 0.17 skin punctures (95% CI −0.08 to 0.41) was not statistically significant (*p* = 0.17).

The mean perceived difficulty score reported for the cross-sectional approach was 3.97 out of 10 (95% CI 3.10 to 4.83). The mean perceived difficulty for the longitudinal approach was 3.93 out of 10 (95% CI 3.21 to 4.65). The difference in mean perceived difficulty of 0.04 out of 10 (95% CI −0.95 to 1.01) was not statistically significant (*p* = 0.95).

All the results are summarised in Table 1.

**Table 1:** Table to show the mean scores and 95% confidence limits for each of the three variables we measured, sorted by approach

	Cross-sectional	Longitudinal
Mean no. of skin punctures	1.13 ± 0.19	1.30 ± 0.22
Mean time (seconds)	45.1 ± 15.8	52.8 ± 16.2
Mean difficulty (out of 10)	3.97 ± 0.87	3.93 ± 0.72

The mean score recorded in each box was generated from the 30 individual scores collected for that condition, as each subject attempted each condition once (i.e. *n* = 30 for each box)

**Fig. 3:**
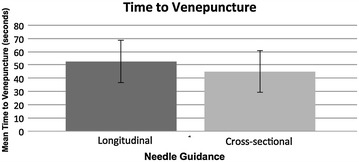
Graph showing the mean time required to achieve successful venepuncture for each of the two approaches. *Error bars* represent the 95% confidence limits of the means

**Fig. 4:**
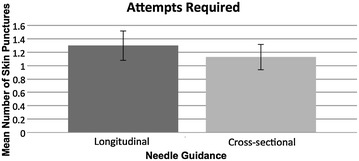
Graph showing the mean number of skin punctures required to achieve successful venepuncture for each of the two approaches. *Error bars* represent the 95% confidence limits of the means

**Fig. 5:**
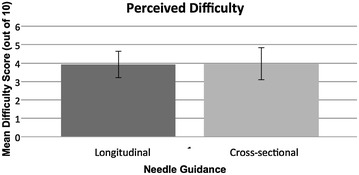
Graph showing the mean perceived difficulty rating for each of the two approaches. *Error bars* represent the 95% confidence limits of the means

For graphical representations of the results, see Figs. 3, 4 and 5.

## Discussion

Since the first emergency department observational study of the use of ultrasound to establish peripheral vascular access in 1999, [5] the availability of both ultrasound equipment and the ultrasound expertise of emergency physicians has expanded considerably. In many emergency departments, ultrasound is being increasingly utilised to obtain PIV access in patients where attempts using the traditional landmark-and-palpation technique have failed [2].

Ultrasound-guided PIV cannulation is also being utilised in other settings such as intensive care units [13]. Use of ultrasound provides a very real alternative to more invasive approaches such as central venous access, [6, 13] external jugular vein, [14] venous cutdown or intraosseous access. In addition, ultrasound-guided PIV cannulation has been shown not to pose a significant infection risk [15].

With regard to the efficacy of ultrasound-guided PIV cannulation over a traditional landmark-and-palpation technique, the literature is conflicting. Whist Constantino found that ultrasound was more successful than traditional techniques [16], not all the literature is positive. Several studies have failed to show any advantage of ultrasound-guided peripheral vascular access over a traditional technique [17, 18]. A study in children under 10 years also failed to show any statistical advantage of ultrasound over a traditional approach [19].

Although ultrasound-guided PIV cannulation has not been shown to be better than traditional techniques in all patients, there is evidence to support its use in difficult patients or where traditional techniques have failed. The international evidence-based recommendations on ultrasound-guided vascular access from 2013 recommended that, “Use of ultrasound should be taken into consideration for any kind of peripheral intravenous line when difficult access is anticipated” [20]. They also recommend the use of the longitudinal approach but accept that it is more challenging than the cross-sectional approach. The longitudinal approach may be safer by allowing better needle tip visualization and thereby minimising posterior wall puncture [8]. With ultrasound-guided central venous access, it can be difficult to distinguish between the vein and the artery in longitudinal view, with a greater risk of arterial puncture with the longitudinal approach. The cross-sectional approach allows identification of not just the vessel to be punctured (the vein) but also the vessel to be avoided (the artery). This visualisation of both vessels is lost with a longitudinal approach.

There is currently no consensus as to whether a longitudinal or a cross-sectional approach is superior. In a single study Mahler showed that the cross-sectional approach was slightly faster, but failed to demonstrate any further benefit over the longitudinal approach [21].

How to teach ultrasound-guided PIV cannulation is less well covered in the literature. After a short training package, perceived difficulty of obtaining peripheral vascular access was reduced in a cohort of emergency nurses [4]. A study involving utilisation of ultrasound to aid PIV access learning by medical students using just the short-axis approach showed no difference in terms of obtaining PIV access, but the ultrasound group did perceive the experience as easier and felt they had gained more knowledge of the mechanics of placing an IV cannula [9].

There has been some focus on how best to teach this skill and also which approach is best (cross-sectional versus longitudinal). Blaivas et al. [7] found a clear superiority of the cross-sectional approach over the longitudinal approach in terms of time taken from placing the ultrasound probe and needle to successful vascular access. Interestingly, our study generated some quite different results, and failed to show a difference despite our sample size being almost twice that used by Blaivas and colleagues. (30 subjects, compared to 17 subjects).

Our study has a number of limitations that should be acknowledged. Firstly, our subjects were required to access simulated vessels in an ultrasound phantom, as opposed to the real vessels in a living human patient. The task of aspirating fluid from a model under ultrasound guidance is undoubtedly easier than taking real blood from a patient, as our subjects did not have to attend to the model’s concerns or discomfort during the procedure. Additionally, the deep peripheral veins in real patients are unlikely to be as straight as the vessels in our inanimate phantom. Acquiring and maintaining an adequate longitudinal view of a tortuous vein in a real patient is likely to be considerably more difficult than obtaining a good longitudinal view in our model. As such it is difficult to extrapolate our findings to clinical practice, and this study does not provide evidence of benefit of one approach over the other in clinical practice. The findings are limited to the training of novices using a phantom model.

It should also be noted that our study measured only whether our subjects could achieve venepuncture, while in reality the insertion of a PIV cannula requires an additional step in which the catheter is inserted into the vessel following venepuncture. We appreciate that venepuncture does not include all the difficulties of cannulation, for example, confirmation of patency and the increased risk of puncturing of the posterior wall. However, as the purpose of ultrasound is to guide the user in locating the vessel rather than the technique of cannulation, we considered it an adequate proxy.

This study has demonstrated that, in novice ultrasound users, neither the longitudinal nor transverse approach showed a clear superiority when ultrasound-guided venepuncture was attempted in a model. More research is needed to definitively answer the question of whether the cross-sectional or longitudinal approach is more effective when teaching this skill to novice ultrasound users. Future researchers may wish to have subjects attempt the technique on human subjects to produce results that translate clearly to real clinical practice. However, considering the clear benefits of peripheral venous access (as demonstrated in other studies), the most pertinent point may be to increase teaching of ultrasound-guided peripheral venous access in general, regardless of approach [2, 3]. Until such times as this question is resolved, we believe that both the longitudinal and cross-sectional approaches have value, and that both techniques should be included when developing a training programme to teach this useful clinical skill.

## Additional files

**Additional file 1.** Blue Phantom ultrasound model.

**Additional file 2.** Cross-sectional ultrasound image of needle in phantom.

**Additional file 3.** Longitudinal ultrasound image of needle in phantom.

## Abbreviations

PIV: peripheral intravenous
IV: intravenous
