# Supplementary figures and images for: A randomised crossover study to compare the cross-sectional and longitudinal approaches to ultrasound-guided peripheral venepuncture in a model
Source: Crit Ultrasound J. 2017 Apr 3;9:9. doi: 10.1186/s13089-017-0064-1 (PMC5376999; doi:10.1186/s13089-017-0064-1)

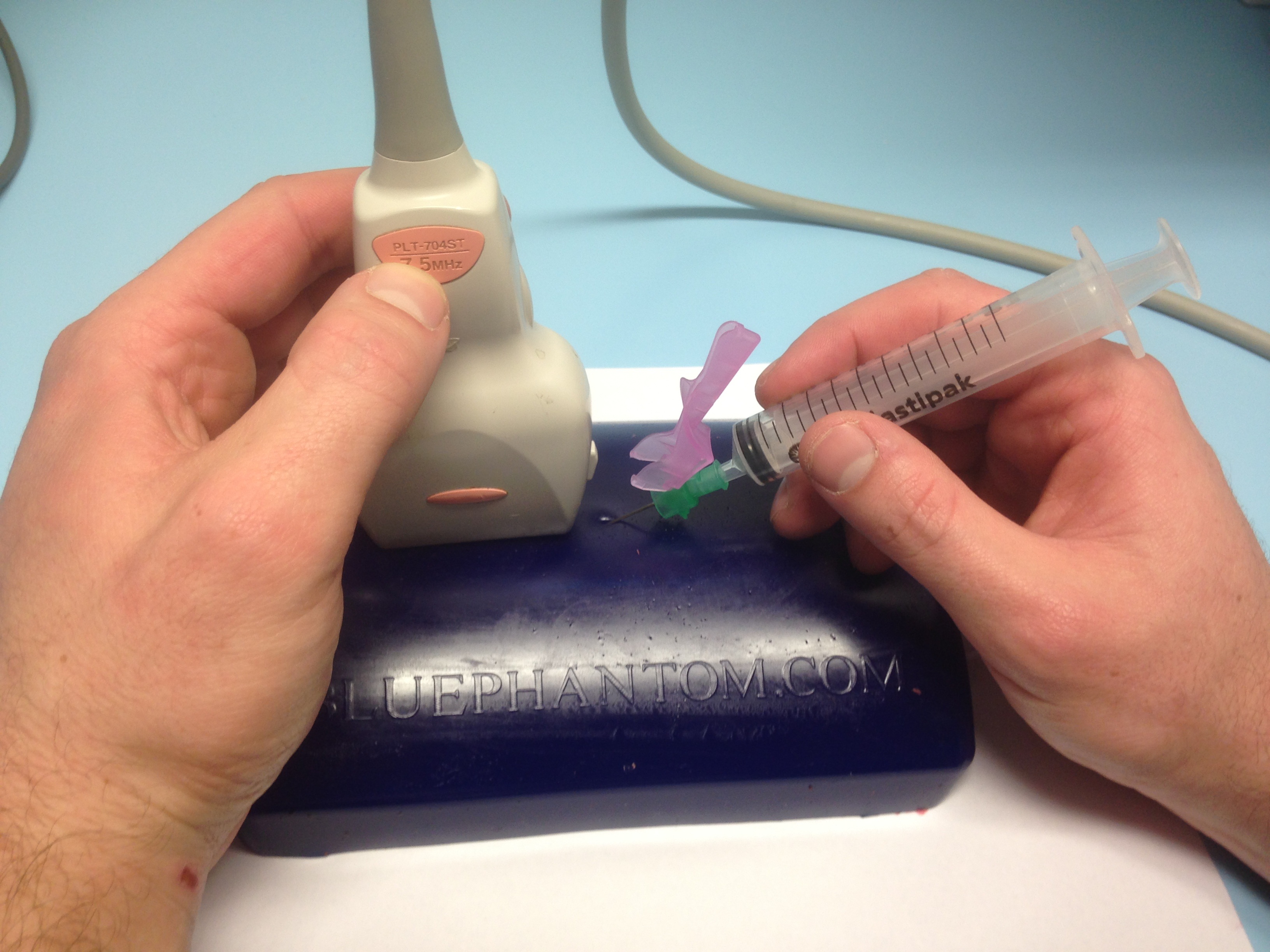

Supplement: Supplementary file 1 — Additional file 1. Blue Phantom ultrasound model. [file 13089_2017_64_MOESM1_ESM.jpg]

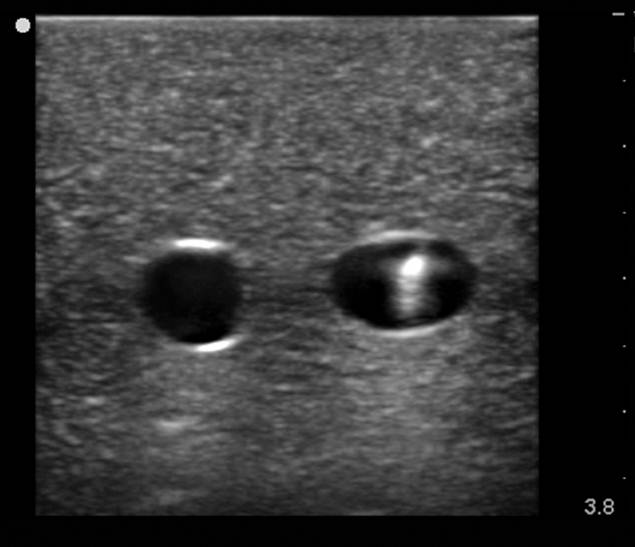

Supplement: Supplementary file 2 — Additional file 2. Cross-sectional ultrasound image of needle in phantom. [file 13089_2017_64_MOESM2_ESM.jpg]

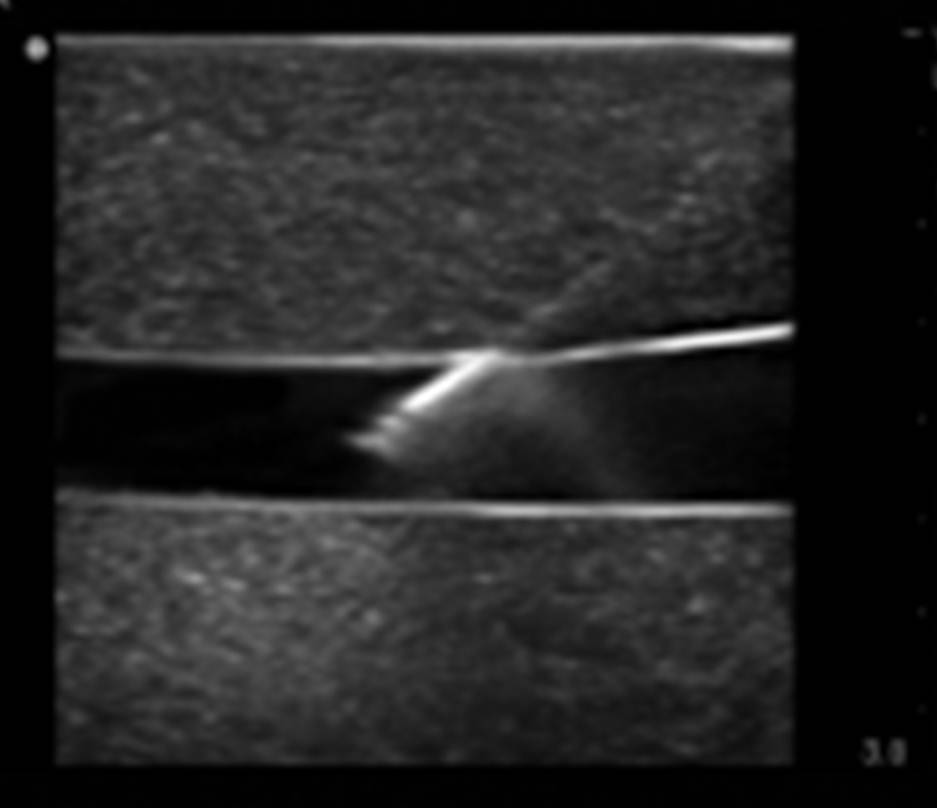

Supplement: Supplementary file 3 — Additional file 3. Longitudinal ultrasound image of needle in phantom. [file 13089_2017_64_MOESM3_ESM.jpg]
